# Quantifying implementation strategy and dissemination channel preferences and experiences for pain management in primary care: a novel implementer-reported outcome

**DOI:** 10.1186/s43058-022-00378-z

**Published:** 2022-12-09

**Authors:** Laura Ellen Ashcraft, Deborah J. Moon, Jessica S. Merlin, Shaun M. Eack, Shari S. Rogal

**Affiliations:** 1 Center for Health Equity Research and Promotion, Corporal Michael Crescenz VA Medical Center, Philadelphia, PA USA; 2 School of Social Work, University of Pittsburgh, Pittsburgh, PA USA; 3 Division of General Internal Medicine, School of Medicine, CHAllenges in Managing and Preventing Pain (CHAMPP) clinical research center, University of Pittsburgh, Pittsburgh, PA USA; 4 Department of Psychiatry, School of Medicine, University of Pittsburgh, Pittsburgh, PA USA; 5 Departments of Medicine and Surgery, University of Pittsburgh, Pittsburgh, PA USA; 6 Center for Health Equity Research and Promotion, VA Pittsburgh Healthcare Center, Pittsburgh, PA USA

**Keywords:** Measurement, Implementer, Concordance, Agreement, Practitioner, Clinician

## Abstract

**Background:**

Precision implementation science requires methods to evaluate and select implementation strategies. This study developed and evaluated a novel measure of concordance between current and preferred dissemination channels (DC) and implementation strategies (IS) to guide efforts to improve the adoption of evidence-based management strategies for chronic pain.

**Methods:**

We conducted a one-time electronic survey of Pennsylvania primary care practitioners (PCPs) about current vs. preferred chronic pain management DC and IS use. Survey items were selected based on preliminary data, the Model for Dissemination of Research, and the Evidence-Based Recommendations for Implementing Change taxonomy of implementation strategies. We used Cohen’s kappa (κ) to assess the agreement between participant-level current and preferred DC/IS. We calculated % preferred minus % experienced for each DC/IS and assessed the equality of proportions to determine whether this difference significantly departed from zero. We categorized DC and IS based on the degree of use and preference, to evaluate alignment.

**Results:**

The current sample included 101 Pennsylvania PCPs primarily in urban (94.06%), non-academic (90.10%) settings who self-identified as mostly female (66.34%) and white (85.15%). The greatest difference between preferred and experienced DCs, or “need,” was identified by participants as workshops, clinical experts, seminars, and researchers. Similarly, participants reported the greatest IS gaps as multidisciplinary chronic pain workgroups, targeted support for clinicians, and a chronic pain clinical champion. Participating PCPs had moderate DC concordance (kappa = 0.45, 95% CI = 0.38–0.52) and low IS concordance (kappa = 0.18, 95% CI = 0.13–0.23). DC and IS concordance were both greater than that expected by chance. We further identified well-aligned DC and IS, including professional organizations, briefs, and guidelines.

**Conclusion:**

We identified a novel implementer-reported outcome of dissemination channel and implementation strategy concordance that allows implementation scientists to quantify the magnitude of the gap between the current and preferred experience of implementers. This quantitative measure can help with the selection and evaluation of dissemination channels and implementation strategies. Future research should leverage this measure to understand the degree to which preference concordance influences clinical outcomes and performance.

**Supplementary Information:**

The online version contains supplementary material available at 10.1186/s43058-022-00378-z.

Contributions to the literature ●Research often describes the experiences of implementers; however, we often do not know the degree of agreement between current and preferred experiences.

●This proof-of-concept study describes a novel implementer-reported outcome which quantifies the degree of agreement between what implementers currently experience and their preferred dissemination channels and implementation strategies.

●Quantifying implementer concordance is a first step toward supporting the dissemination and implementation of evidence-based practices.

## Background

The US Precision Medicine Initiative was created to focus biomedical efforts and leverage scientific advances to improve health [1–3]. However, precision medicine remains to be realized in routine care [4]. Dissemination and implementation science (D&I) aims to advance these efforts by introducing methods to disseminate and implement evidence-based practices. Within D&I, there is a parallel movement toward precision [4], which includes using data and empirical methods to inform selection of dissemination channels and implementation strategies.

Dissemination channels describe *how* a message is communicated from the source of the information to the target audience [5–7]. Dissemination channels range from social media to one-on-one meetings, and also include workshops and seminars [5]. The challenges associated with the misalignment of dissemination channels are widely acknowledged (e.g., communicating research findings only through the peer-reviewed literature) and can affect both short and long-term outcomes [5].

Implementation strategies are defined as methods to enhance the adoption and maintenance of an evidence-based practice or program [8]. These include diverse techniques, such as changing infrastructure, using data, or reaching out directly to patients [8, 9]. Historically, implementation scientists have relied on qualitative data to identify implementation barriers and facilitators to inform implementation strategy selection. Recent efforts have sought to make this process more empirical by quantifying strategies and implementation determinants [10, 11].

One area that has been less explored is stakeholder preferences for how they receive information (dissemination channels) and how they would like to translate evidence into practice (implementation strategies). Prior work has sought to understand dissemination channel preferences among legislative policymakers and teachers [12–14]. However, this work has not quantified how current dissemination channels and implementation strategies align with stakeholder preferences. This alignment may be an important, stand-alone predictor of implementation success and the effectiveness of dissemination.

Cohen’s kappa (κ) reports a co-efficient of agreement while factoring for agreement by chance [15]. Often Cohen’s kappa (κ) is applied with two raters who assess at the same point in time or with one rater at different times [16]. We developed and tested this novel measurement within the context of chronic pain management in primary care. We chose chronic pain because it affects the physical, social, psychological, and economic well-being of millions of people worldwide [17, 18] and is typically managed in primary care settings [19, 20]. However, constantly evolving guidelines contribute to therapeutic uncertainty and overreliance on potentially harmful analgesics and untreated chronic pain for patients [17, 19, 21, 22].

To address this methodological and topical gap, the goal of the current study was to develop a method to quantify concordance between preferences and experiences around dissemination and implementation.

## Methods

### Study design

To test the use of Cohen’s kappa (κ), we employed a cross-sectional survey design using a convenience sample of primary care providers (PCPs). We chose to focus on how PCPs learn about evidence-based chronic pain management and the ways in which they are supported to translate the evidence into practice. We followed quality reporting methods for cross-sectional studies using the strengthening the reporting of observational studies in epidemiology (STROBE) checklist (see Online Supplement A).

### Survey development

We developed a list of dissemination channels and implementation strategies based on prior exploratory qualitative research with Pennsylvania PCPs (*Ashcraft et al., under review*) and the Model for Dissemination of Research [5]. We began with a list of implementation strategies listed in previous work (*Ashcraft et al., under review*) and then iteratively integrated themes from the nine Evidence-Based Recommendations for Implementing Change (ERIC) clusters [9]. We then tailored each strategy to be relevant to chronic pain management in primary care settings. The lists of potential channels and strategies were reviewed by the study team and tailored to include those channels and strategies that were deemed to be the most relevant to the clinical context. The survey was iteratively revised and then piloted with clinicians, whose feedback was integrated to improve readability, clarity, and acceptability (in terms of length and organization).

The final survey included 34 questions about currently used dissemination channels for learning about chronic pain management and dissemination channels preferred in an ideal world. We then asked about current and ideal world preferences for strategies to implement evidence-based chronic pain management. Possible responses were yes/no. We also requested information about professional training and clinic context questions (See Online Supplement B for the study survey).

### Participant eligibility and recruitment

This convenience sample included practitioners treating chronic pain within a given policy context. For this reason, we recruited Pennsylvania physicians, nurse practitioners, and physician assistants who work in outpatient primary care settings. For inclusion criteria, potential participants must (1) be a physician, physician assistant, or nurse practitioner; (2) practice in an outpatient primary care setting in Pennsylvania; (3) not practice more than 50% in a federally qualified health center (FQHC); and (4) not practice in pediatric primary care. We excluded PCPs working primarily in FQHCs because we hypothesized that FQHCs provide targeted healthcare support for marginalized populations. We excluded PCPs who work primarily in pediatric healthcare settings, as approaches to treat chronic pain are distinct in children. We distributed a one-time, electronic survey to Pennsylvania PCPs. Participants were eligible to enter sweepstakes to win one of eight $100 Amazon gift cards.

### Analysis

Analyses were conducted in *Stata* version 15 [23]*.* The analytic flat file and analytic code (*Stata .do file*) are available as Online Supplements C and D, respectively. We first used descriptive statistics to understand the frequencies of dissemination and implementation preferences. We then examined alignment for each dissemination channel and implementation strategy across the sample by assessing the percentage point difference between reported current and ideal-world preferences.

### Percentage point difference to assess sample-level channel and strategy agreement

We tested to see if the percentage point difference between current and ideal world preferences for each dissemination channel and implementation strategy was statistically different from zero using the *prtest* in *Stata* to assess equality of proportions [24]*.* This information provides additional insights into the degree to which specific channels and/or strategies are currently used and/or preferred by participants.

### Cohen’s kappa (κ) to assess implementer preference alignment

Cohen’s kappa (κ) was employed to evaluate preference concordance for dissemination channels and implementation strategies. We then summarized Cohen’s kappa (κ) at both the sample and individual levels. The following describes our novel use of Cohen’s kappa (κ) to assess implementer preference alignment and how we tested this method in a sample of Pennsylvania primary care practitioners (PCPs). We consider the terms preference, alignment, and concordance to be interchangeable.

Cohen’s kappa (κ) is generally used to assess the degree of agreement between ratings and ranges from −1 to 1 with 0 indicating agreement no different from that of chance [15]. Cohen’s kappa (κ) is not a raw percentage (i.e., the percent agreement), rather kappa (κ) is a coefficient of agreement which incorporates the possibility of agreement by chance such that a kappa (κ) score equal to that of chance is 0 whereas a percent agreement due to chance is 50% [15]. Cohen’s kappa (κ) is often used to evaluate the degree of agreement between two raters evaluating the same information at the same time or the degree of alignment between one rater at two different points in time (e.g., pre- and post-test) [16].

As with most statistical tests, there are several underlying assumptions with kappa (κ). Cohen’s original assumptions require that (1) the outcome variables are nominal or categorical, (2) the units are independent, and (3) the raters function independently [15]. We used nominal outcome variables (i.e., selected or not selected channels or strategies). Our response options, or units, were independent of one another (i.e., each dissemination channel or implementation strategy), and the raters functioned independently.

Cohen’s kappa (κ) can be, and is often, used to evaluate the consistency of one rater across two points in time [16]. The innovation in our approach was using Cohen’s kappa (κ) to view the implementer as the single person with two data points, separated not in time, but rather into current experience versus ideal/preferred experience. Using Cohen’s kappa (κ), we thus assessed the degree of agreement to which preferences match experiences. Rather than focusing on two times, the comparison was used to evaluate reality vs. preferences, collected at a single point in time. Cross-sectional data collection cannot be used to evaluate *changes in preference over time*. Instead, we evaluated the degree of concordance between what is currently experienced and what would be preferred in an ideal state, by person, at a single time point.

### Calculated Cohen’s kappa (κ)

Table 1 shows an individual, fictitious example, to illustrate how we applied Cohen’s kappa (κ) to assess agreement. A list of dissemination channels was evaluated as follows: “Currently, how do you learn about managing chronic pain? *Select all that apply.*” Each response was coded as 1 (selected) or 0 (not selected). Next, the implementer is asked, “In an ideal world, how would you learn about managing chronic pain? *Select all that apply.*” Again, the response is converted into a bivariate 1 or 0.

**Table 1:** Example of how Cohen’s kappa is used to assess concordance

	Current	Preferred
Colleagues	1	0
Your own clinical experience	1	0
Patients	0	0
Professional organizations	0	1
Researchers	0	1
Clinical experts	1	0
Pharmaceutical representatives	0	1
Primary peer-reviewed literature (e.g., PubMed)	0	1
Online peer-reviewed clinical resources (e.g., UptoDate)	0	0
Email listserv	1	0
Practice Briefs or Practice Guidelines	1	0
Annual conferences	0	1
Seminars at my clinic/institution (e.g., grand rounds; case conference)	0	0
Web-based continuing education modules	1	0
Workshops on specific intervention (e.g., CBT, Yoga)	1	1
Main-stream media (e.g., NPR, CNN, FoxNews)	0	0
Blogs (e.g., Tumblr, Wordpress)	1	0
Social media (e.g., Facebook, Twitter, Reddit)	1	1
Podcasts	1	0
Other	0	1
None of these	0	0
Agreement, *n* (%)	7 (33)	
Disagreement, *n* (%)	14 (66)	
Cohen’s kappa (κ)	−.33	

Table 1 shows an example of how the data were structured to assess the degree of concordance or agreement between what a fictional provider is currently experiencing and what they would prefer to experience in an ideal scenario with one indicating yes and zero indicating no. Percent agreement is the number and percent of total response options the fictional respondent agreed between current and preferred. Disagreement is the number and percent of the total response options the fictional respondent did not provide the same answer for both current and preferred. Cohen’s kappa (κ) was calculated to describe the level of actual concordance for the fictional provider

Table 1 also provides an example to illustrate how we calculated percent agreement, disagreement, and expected agreement by chance. For this example, 7 (33%) dissemination channels met agreement. Of these seven, two were being currently used and preferred in an ideal world (e.g., workshops) and five were not being used and not preferred as dissemination channels for evidence-based chronic pain management (e.g., online peer-reviewed clinical resources). Conversely, there was discordance about preferences and experiences for 14 (66%) of the dissemination channels. For eight channels, the respondent reported experiencing a dissemination channel that they did not prefer (e.g., clinical experts); for six channels, respondents reported not experiencing a dissemination channel and would like to experience it in an ideal world (e.g., professional organizations). Using these data, Cohen’s kappa (κ) was then calculated to assess the degree of agreement between dissemination strategies currently experienced and preferred in an ideal world. In this example, Cohen’s kappa (κ) is calculated to be −0.33.

### Interpreting Cohen’s kappa (κ)

Among several accepted approaches to interpret Cohen’s kappa (κ) [16, 25], we chose to use a seven-category schema, to allow for more detailed categorization. This schema uses 0-0.09 indicating no difference from chance, 0.10–0.20 indicating slight agreement, 0.21–0.40 indicating fair agreement, 0.41–0.60 indicating moderate agreement, 0.61–0.80 indicating substantial agreement, 0.81–0.99 indicating near perfect agreement, and 1 signifying perfect agreement, with the inverse indicating disagreement [16, 25]. Using this categorization approach, the C﻿ohen’s kappa (κ) of −0.33 in our fictitious example (Table 1) would be interpreted as “fair disagreement” between current and preferred dissemination channels.

### Sample size calculation

Sample size and power calculations for Cohen’s kappa (κ) are typically used to identify the number of cases, or patients, needed to be assessed between the two reviewers in order to achieve a pre-specified power threshold or confidence interval [26]. In our application of Cohen’s kappa (κ), this would look like a single participant needing to rate over one hundred dissemination channels and implementation strategies. Instead, we used Cohen’s kappa (κ) as a descriptive indicator of individual level agreement and aggregated that agreement or series of Cohen’s kappa’s (κ) across a convenience sample. For this application, it was not appropriate to calculate the sample size or conduct a post hoc power analysis.

## Results

### Sample description

We conducted a one-time electronic survey of a convenience sample of 101 Pennsylvania PCPs between January and May 2021. We made 380 documented contacts with professional practice organizations, provider groups, on social media, and to available resources at the University of Pittsburgh (e.g., Pitt+Me, PaTH Network). In total, 252 people responded to an invitation to complete the survey with 136 (54%) meeting the inclusion criteria. Sixty-nine people who attempted to take the survey reported learning about the study from UPMC Community Medicine, Inc. primary care practice. Of the 136 who met the inclusion criteria, 115 (85%) people completed the full survey. In the first month of data collection (January 2021) an error in the survey led to exclusion of an additional 14 respondents,^1^ leaving 101 respondents in the final analytic sample. The median time to complete the survey was about 8 min (IQR=6–9 min). The short completion time, high response rate, and completeness of data indicate that the survey length is perceived as acceptable by participants and is feasible (or practical) as a method by which to collect these data.

Participants were mostly non-Hispanic white (*n*=86; 85.2%) and self-identified as female (*n*=67; 66.3%). Most participants had MDs (*n*=63; 62.4%) and practiced on average 3.36 days per week (SD=1.41) in the clinic with an average of 12.29 years of experience (SD=10.70). Full sample characteristics are presented in Table 2.

**Table 2:** Participant characteristics

Variable name	Missing	N	%	M	SD
Race	2	99			
White		86	85.1		
Ethnicity^1^	0	101			
Hispanic		5	5.0		
Latino		1	1.0		
Gender (female)	3	67	66.3		
Profession (physician)	1	71	70.3		
MD		63	62.4		
DO		8	7.9		
CRNP		15	14.8		
PA-C		14	13.9		
Days per week in clinic	3	98		3.36	1.4
Years of experience	3	98		12.3	10.7
Estimated % patients with chronic pain	0	101		31.3	18.5
Setting Urban	2	95	94.1		
Part of health system	1	93	92.1		
Non-academic setting	0	91	90.1		
Teaching		77	76.2		
Research		12	11.9		
Accepts all insurance types	1	92	91.1		
Commercial	1	95	94.1		
Medicare	1	95	94.1		
Medicaid	1	96	95.0		
None		0	0		
Other	1	4	4.0		

*Sample size: 101;*^1^The term Hispanic refers to people from Spanish-speaking Latin American countries. The term Latino refers to people who live in Latin America in countries colonized by Spain or Portugal and is inclusive of non-Spanish speaking countries (e.g., Brazil)

Table 2 describes the participant characteristics for the sample of Pennsylvania primary care providers. We report race and ethnicity in adherence with funding requirements from the US National Institutes of Health

The following describes (1) preference alignment at the channel or strategy level; (2) the degree of agreement between preferred and actual channels and strategies for the overall sample (“sample Kappa”); and (3) individual participant concordance.

### Dissemination channel preferences and alignment with experiences

The most frequently endorsed preferred dissemination channels (“in an ideal world”) were online clinical resources, seminars, clinical experts, colleagues, peer-reviewed literature, briefs/guidelines, and conferences. However, the most frequently used dissemination channels were clinician experience, colleagues, and online clinical resources (Table 3).

**Table 3:**
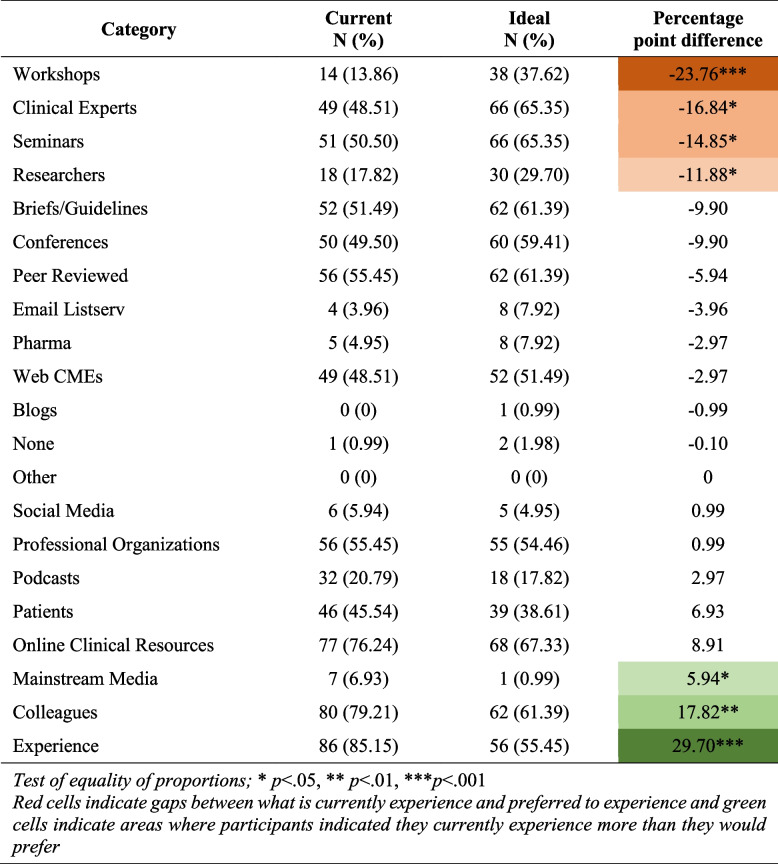
Description of dissemination channels

Test of equality of proportions; * *p*<.05, ** *p*<.01, *** *p*<.001

Red cells indicate gaps between what is currently experienced and preferred to experience and green cells indicate areas where participants indicated they currently experience more than they would prefer

We then compared the percentage point differences between channels that were used vs. preferred. The channels that were preferred but not used in the real world were: workshops (−23.76, *p*<0.001), clinical experts (−16.84, *p*<0.05), seminars (−14.85, *p*<0.05), and researchers (−11.88, *p*<.005). Conversely, experience (29.70, *p*<0.001) and colleagues (17.82, *p*<0.01) were frequently used but not preferred.

We interpret non-significant percentage point difference between current and preferred as an indication of dissemination channels that were well-aligned. These included conferences, professional organizations, and online clinical resources, to name a few. Most dissemination channel preferences and those that were used matched.

### Implementation strategy preferences and alignment with experiences

The most frequently used implementation strategies were consulting experts and tailoring treatments. However, the most preferred implementation strategies were using workgroups, conducting chronic pain education, conducting needs assessments, tailoring treatments, consulting experts, engaging patients and families, providing targeted support, and using data to inform care (Table 4).

**Table 4:**
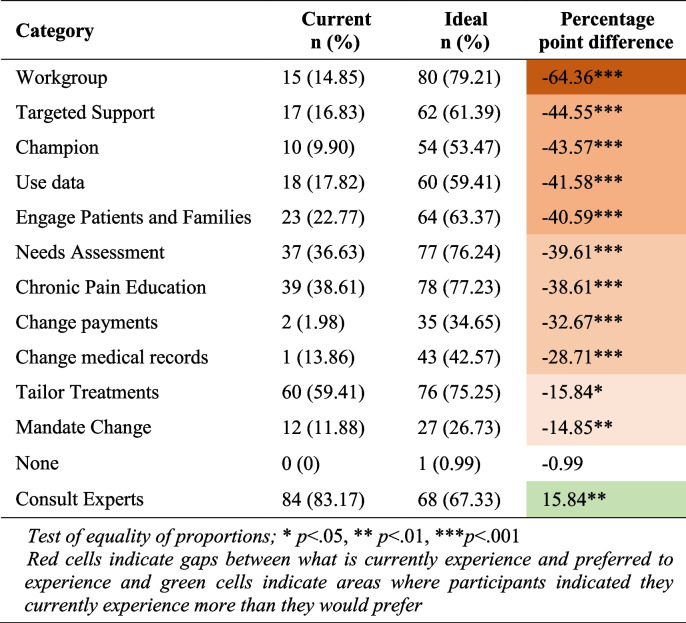
Description of implementation strategies

Test of equality of proportions; * *p*<.05, ** *p*<.01, *** *p*<.001

Red cells indicate gaps between what is currently experienced and preferred to experience and green cells indicate areas where participants indicated they currently experience more than they would prefer

The largest gaps in preference alignment among this sample of Pennsylvania PCPs were for the following strategies: “*Develop an interdisciplinary workgroup to address chronic pain*” with a percentage point difference of −64.36 (*p*<0.001); “*Provide targeted support for clinicians treating chronic pain*” (−44.55, *p*<0.001), “*Develop a chronic pain champion in clinic*” (−43.57, *p*<0.001), “*Use data to inform care*” (−41.58, *p*<0.001), and “*Directly engage patients or families in the process of quality improvement around chronic pain management*” (−40.59, *p*<0.001). All implementation strategies except “*consult experts*” (15.84, *p*<0.01) and “*none*” (−0.99) had statistically significant gaps between what was used and preferred.

In contrast to dissemination channels, there was poor alignment between ideal/preferred implementation strategies and those that were used by implementers. Many implementation strategies were wanted more than currently experienced, such as using workgroups, engaging patients and families, and chronic pain education.

**Fig. 1:**
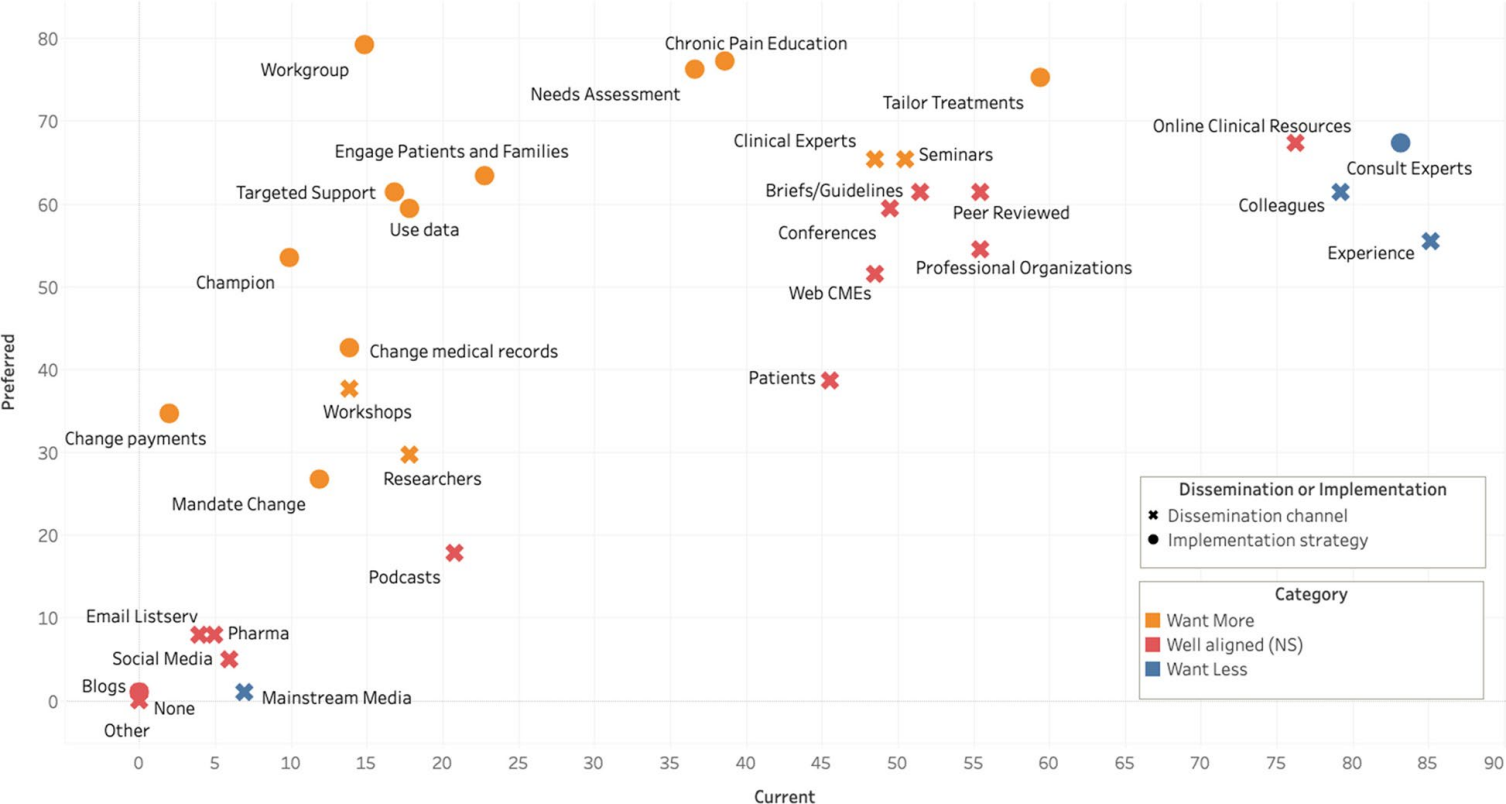
Dissemination channel and Implementation strategy current experiences and preferences among PCPs

Fig. 1 displays both dissemination channel and implementation strategy data visually to identify the areas of greatest difference between preferences and real-world use. An optimized and interactive Fig. 1 is available online through Tableau Public (link here).

### Sample kappa (κ): concordance between individual preferences and experiences

Cohen’s kappa (κ) was used to assess each participant’s overall preference alignment for both dissemination channels and implementation strategies (as described above). At the sample level, participants had an average dissemination concordance (or preference alignment) of 0.45 (SD=0.33; range −0.5–1; 95% CI: 0.38–0.52) and average implementation concordance of 0.18 (SD=0.26; range −0.41–1; 95% CI: 0.13–0.23).

**Fig. 2:**
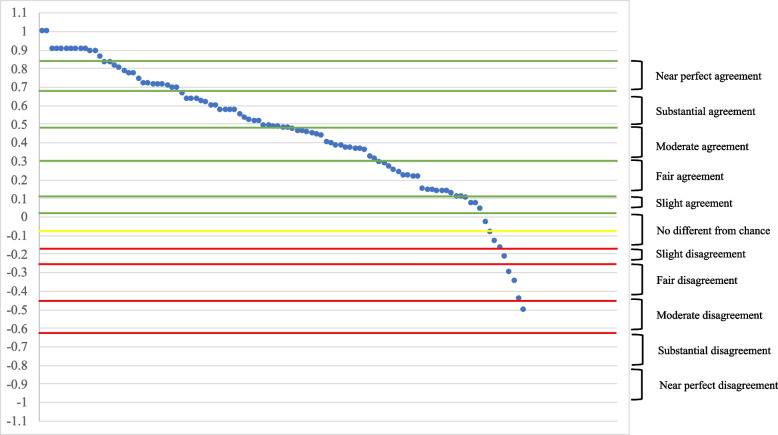
Dissemination concordance of Pennsylvania primary care providers as calculated by Cohen’s kappa (κ)

Next, we describe Cohen’s kappa (κ) results at the individual level. As stated above, we used the seven-level categorical description of Cohen’s kappa (κ) to understand the distribution across the sample for both dissemination concordance and implementation concordance. Most participants had a fair or higher agreement on dissemination channels (see Table 5), indicating that they were generally receiving information through their preferred channels. Fig. 2 illustrates the distribution of individual Cohen’s kappa (κ) scores for dissemination channels.

**Table 5:** Categorical concordance

Cohen’s kappa (κ)	Categorical interpretation	Dissemination concordance *n* (%) [M]	Implementation concordance *n* (%) [M]
−1	Perfect disagreement		
−0.81 to −0.99	Near perfect disagreement		
−0.61 to −0.80	Substantial disagreement		
−0.41to −0.60	Moderate disagreement	2 (2.0) [−0.47]	
−0.21 to −0.40	Fair disagreement	3 (3.0) [−0.29]	6 (5.9) [−0.30]
−0.10 to −0.20	Slight disagreement	2 (2.0) [−0.15]	8 (7.9) [−0.15]
−0.09–0.09	No different from chance	5 (4.9) [0.01]	24 (23.8) [0.03]
0.10–0.20	Slight agreement	10 (9.9) [0.13]	23 (22.8) [0.15]
0.21–0.40	Fair agreement	20 (19.8) [0.31]	25 (24.7) [0.29]
0.41–0.60	Moderate agreement	24 (23.8) [0.51]	9 (8.9) [0.50]
0.61–0.80	Substantial agreement	19 (18.8) [0.70]	3 (3.0) [0.65]
0.81–0.99	Near perfect agreement	14 (13.9) [0.88]	1 (1.0) [0.85]
1	Perfect agreement	2 (2.0) [1.0]	2 (2.0) [1.0]

Table 5 shows the seven-level categorization of Cohen’s kappa (κ) at the individual level. Dissemination and implementation concordance were calculated for each participant and categorized. We reported the number of participants, the percent of total, and average for each category

**Fig. 3:**
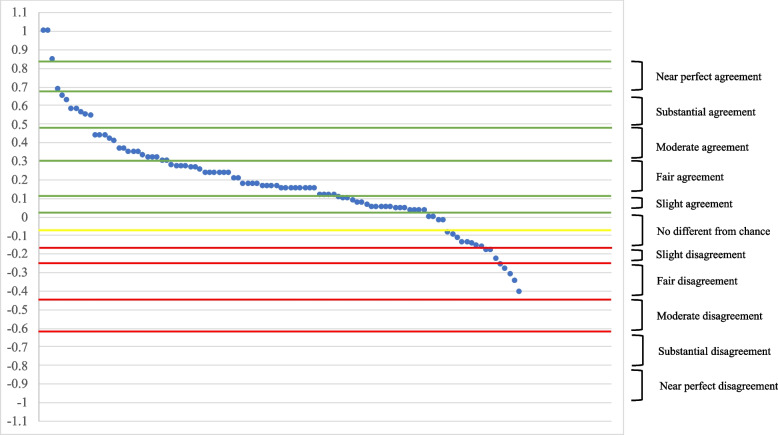
Implementation concordance of Pennsylvania primary care providers as calculated by Cohen’s kappa (κ)

Overall implementation strategy concordance was lower, indicating that participants had lower levels of agreement between current experiences and their ideal world. The highest concentrations of implementation concordance were in the categories of no difference from chance (*n*=24; 23.8%), slight agreement (*n*=23; 22.8%), and fair agreement (*n*=25; 24.8%) (see Table 5). This is also represented in Fig. 3 and again shows that most participants had some but overall low levels of agreement between current experiences and preferred implementation strategies.

## Discussion

The reported data illustrate the feasibility and value of measuring stakeholder (implementer) preferences and comparing these to the real-world use of dissemination channels and implementation strategies. Using a novel application of Cohen’s kappa (κ), we quantified the degree of agreement between currently *experienced* dissemination channels and implementation strategies and *preferred* dissemination channels and implementation strategies. This provided an innovative quantitative approach to engaging stakeholders in developing implementation strategies and selecting dissemination channels to support evidence-based practices in pain management. This approach was feasible and acceptable to implementers, as measured by the high response rate, short completion time, and low rates of missing data.

To test this novel method, we examined the degree of dissemination and implementation preference alignment among Pennsylvania PCPs by soliciting information about how they currently learn about evidence-based chronic pain management and how they would prefer to learn about it in an ideal world (dissemination concordance) and also what the strategies they currently use put evidence-based chronic pain management into practice and what strategies they would prefer to use (implementation concordance). Chronic pain was selected because of its ongoing salience and chronic pain management is a high-priority focus with many disparate messages, interventions, and approaches being trialed [17, 18, 25, 26].

We identified both dissemination channel and implementation strategy gaps in this sample. PCPs requested more workshops, dissemination by clinical experts, and seminars to learn about evidence-based chronic pain management (i.e., dissemination channels). Participants identified top strategies including the use of multidisciplinary workgroups, targeted support, and the role of champions.

In this sample, there were more gaps between current and preferred implementation strategies than dissemination channels. At the sample level and on average, both dissemination concordance (M=0.45; SD=0.33; range −0.5–1; 95% CI: 0.38–0.52) and implementation concordance (M=0.18; SD=0.26; range −0.41–1; 95% CI: 0.13–0.23) were higher than that anticipated by chance. When describing individual degrees of preference alignment (i.e., concordance) categorically, most participants had fair, moderate, or substantial degrees of agreement for dissemination concordance. In contrast, most participants’ implementation concordance was no different from chance, slight, or fair agreement. This indicates that PCPs receive information in ways that aligned with their preferences. We would expect that implementation strategies, which are less in the control of the individual provider, would be less concordant. Moreover, it is unclear if the goal should be perfect or near perfect agreement or if an agreement greater than that expected by chance is sufficient. Future research should assess how discordance is associated with process and clinical outcomes.

PCPs reported that they preferred more mandated changes. This was surprising, given that top-down mandates are usually viewed unfavorably and have been associated with worse performance in terms of clinical care in other contexts [8]. However, this preference may reflect the clinical area that we assessed and not a universal preference for more regulations or mandates. Rather, this may indicate practitioners’ desire for clarity, given mixed and changing messaging around chronic pain management and specifically opioid prescribing. Additionally, the participants may perceive that mandates coincide with organizational or structural changes. Future work could examine whether a preference for mandates is associated with performance on objective care measures.

Other methods exist to elicit preferences and may be considered as alternatives to the current approach. For example, Discrete Choice Experiments use an experimental approach to solicit a series of choices embedded within a set of attributes [27]. Recent advances have made this approach more attainable by using survey technology (i.e., Qualtrics) and are a robust way to assess preferences [27]. Alternatively, best-worst scaling builds on Discrete Choice Experiments to more clearly identify preferences [28]. However, both approaches are limited by an inability to compare the current experience to preferred experiences [28] and therefore are not well-suited to understand preference alignment.

### Limitations

There are several limitations to this otherwise novel study. First, it is unclear the extent to which the questions were interpreted as intended. To mitigate this issue, we developed the questions with clinicians and used example scenarios. For example, for the implementation strategy of using champions, we included the definition of a champion, “*Develop a chronic pain champion in clinic (a local clinic member who is passionate about improving chronic pain management)*.” We further developed and refined the survey with clinicians, incorporating their feedback iteratively in our design. Furthermore, we attempted to be responsive to the early misinterpretation of the survey by rewording the options, despite that this required us to eliminate 14 participants. Some confusion could have been caused by the generic focus on evidence-based pain management practices, rather than a single, clearly defined EBP. This provided more breadth but not as much in-depth information about a specific item but may have made questions harder to answer.

Second, we were unable to assess *why* respondents experienced discordance or what the downstream consequences of discordance were. Discordance was lower for dissemination channels, where there is presumably more control about information flow; however, it was unclear why there was any discordance in this area. Furthermore, the implications of misalignment require future study and association with clinical performance. Third, we selected one of several approaches when we interpreted Cohen’s Kappa (κ). Some choose to simply report the raw output, rather than a categorical approach (e.g., −0.33, per our example). However, this can make it difficult to meaningfully interpret the results. However, even a categorical approach requires setting somewhat arbitrary thresholds for levels of agreement, which has been criticized in the literature [25]. To address this concern and for overall data and analytic transparency, we provide the raw data (Online Supplement C). Likewise, we did not assess the knowledge about chronic pain management and thus could not evaluate the associations between concordance and clinical skills (i.e., we could not assess the effectiveness of the dissemination channels for producing more knowledgeable providers).

Our sampling method also had several limitations. This study was cross-sectional by design, with the intent of measuring what was experienced vs. preferred at a single timepoint, so we could not assess preferences over time. The sample was also a small convenience sample and limited to PCPs caring for adults in non-FQHC settings. The purpose of this was to assess a relatively homogenous population of providers in an acceptable fashion, given competing priorities. FQHCs are unique clinical settings, as they receive additional support to provide healthcare to marginalized populations and may not reflect other primary care practice settings. We also excluded PCPs who worked primarily in pediatric populations as chronic pain and chronic pain management are fundamentally different in children. The sample was constrained to a geographic area (Pennsylvania), which further limits the generalizability of the findings. Furthermore, a convenience sample approach also introduces the potential for selection bias.

Finally, our innovation for the third assumption of Cohen’s kappa (κ) regarding the independence of raters. While Cohen’s kappa (κ) has been demonstrated to assess agreement between the same rater at different points in time, we are not aware of other uses of Cohen’s kappa (κ) to assess rater agreement at two *conceptual* points in time (i.e., current and future “ideal” state). Future research may assess this application by comparing the proposed approach with levels of agreement across two points in time such as six months or a year. However, despite these limitations, this proof-of-concept paper provides a novel insight into concordance between what was preferred vs. what was experienced.

### Future directions and potential applications

Cohen’s kappa (κ) is a straightforward way to assess preference alignment among a sample of implementers. While we tested its use in Pennsylvania PCPs, it can easily be employed in other populations and using a range of software (e.g., Microsoft Excel, Google Sheets) that is available in both research and clinical settings.

This measure can be used to inform the selection of dissemination and implementation interventions for implementation trials. This may be a useful tool in the pre-implementation phase of a project to identify dissemination channel gaps experienced by staff and preferred implementation strategies. Interventionists and implementation leaders can then design approaches that align stated preferences with barriers and facilitators to improve the chances of successful implementation.

A necessary next step is to assess the extent to which dissemination and implementation preference alignment are associated with clinical care, knowledge, and implementation success (i.e., does receiving information in a way that clinicians prefer make a difference for patient outcomes?). This measure may function as a determinant of implementation success, wherein we would hypothesize that greater alignment would be associated with improved implementation. Further work may assess this measure over time to understand if changes in the degree of concordance influence behavior change (i.e., if implementation concordance increases are PCPs more likely to utilize evidence-based chronic pain management with their patients?). For dissemination, a potential pathway may indicate that for those with higher levels of concordance there may be higher levels of chronic pain knowledge which may exist because of receiving information in the way a practitioner prefers. For implementation, preference concordance may result in behavior change which may then result in changes in care management and ultimately clinical outcomes. Future work should conduct a series of regression analyses to see if preference concordance is associated with clinical outcomes as a potential target for future behavior change. In doing this, we can quantify the ways in which dissemination channels and/or implementation strategies have a specific influence on outcomes.

Future cognitive interviews may allow us to better understand the clarity of and degree of overlap between the items. This may also help to determine which dissemination channels and implementation strategies are common across clinical areas and implementer populations. Additionally, such interviews could further identify additional channels and strategies to assess as well as the perceived causes for discordance.

Further, we can begin to explore the degree to which some dissemination channels and implementation strategies have potentially disproportionate influence over others. For example, future work could assess the influence of self-reported multidisciplinary workgroup use and the effects of patient adherence to cognitive behavioral therapy for chronic pain management. This could allow implementation scientists and quality improvement work to target specific implementation strategies which are most effective to help patients.

These data speak about perceptions of preferences and real-life experiences of clinicians. However, they do not address the extent to which these preferences and perceptions are “accurate” (i.e., do clinicians prefer the most effective, efficient, scalable, and affordable strategies?) Data regarding the effectiveness of implementation strategies are emerging in the literature, and it is also clear that the most effective strategies are often context dependent. One approach may include using the EASE (effectiveness, affordable, scalability, and efficiency) criteria to evaluate each proposed implementation strategy [29]. Furthermore, strategies may take different forms and functions, regardless of having the common taxonomy. However, triangulating the perceived vs. actual effectiveness of strategies is of ongoing interest to the field.

Finally, future research should explore how aggregate concordance may be an organizational factor and its influence on implementation. Additionally, are there organizational characteristics that are correlated with higher (or lower) levels of concordance. In turn, this may help us to design interventions and implementation approaches that may best meet the needs of implementing clinicians.

## Conclusions

We developed and field tested a novel approach for evaluating the experiences and preferences of clinical implementers, using surveys and Cohen’s kappa (κ) to quantify gaps in dissemination channels and implementation strategies. Future research should examine the relationship between preference alignment and patient outcomes and target specific dissemination channels and implementation strategies which may have a differential effect on implementation.

## Supplementary Information

**Additional file 1.**

**Additional file 2.**

**Additional file 3.**

**Additional file 4.**

## Data Availability

All data and analytic code applicable to this study are included in this published article and its supplementary information files.

## Abbreviations

PCPs: Primary care practitioners
FQHC: Federally Qualified Health Center
ERIC: Evidence-Based Recommendations for Implementing Change
DC: Dissemination channels
IS: Implementation strategies
D&I: Dissemination and implementation

## Footnotes

1 In the original survey format, the dissemination and implementation concordance questions employed a matrix format. Participants were confused about options to select either current or ideal state or both current and ideal state for both dissemination channels and implementation strategies. Due to this confusion, the PI excluded participants from the early phase of the study who had no active (selected) overlap in current and preferred dissemination channels/implementation strategies. Less than one month into survey administration, the survey was changed to separate *select all that apply* questions. This resulted in the exclusion of 14 participants.
