# Supplementary material for: Quantifying implementation strategy and dissemination channel preferences and experiences for pain management in primary care: a novel implementer-reported outcome
Source: Implement Sci Commun. 2022 Dec 9;3:128. doi: 10.1186/s43058-022-00378-z (PMC9733293; doi:10.1186/s43058-022-00378-z)
Supplement: Supplementary file 2 — Additional file 2. [file 43058_2022_378_MOESM2_ESM.docx]

**Online Supplement A: Section of Study Survey**

3.1 Section 1: Learning about Chronic Pain Management

*There are many ways you may learn about chronic pain management and treatment options.*

*Sometimes, you may not be able to find information in a way that works best for you. We want to know more about how you learn about chronic pain and how you wish you could learn about chronic pain.*

3.2 **Currently,** how do you learn about managing chronic pain? *Select all that apply.*

- Colleagues
- Your own clinical experience
- Patients
- Professional organizations
- Researchers
- Clinical experts
- Pharmaceutical representatives
- Primary peer-reviewed literature (e.g., PubMed)
- Online peer reviewed clinical resources (e.g., UptoDate)
- Email listserv
- Practice Briefs or Practice Guidelines
- Annual conferences
- Seminars at my clinic/institution (e.g., grand rounds; case conference)
- Web-based continuing education modules
- Workshops on specific intervention (e.g. CBT, Yoga)
- Main-stream media (e.g. NPR, CNN, FoxNews)
- Blogs (e.g. Tumblr, Wordpress)
- Social media (e.g. Facebook, Twitter, Reddit)
- Podcasts
- Other ______________________________________________
- None of these

3.3 **In an ideal world,** how would you learn about managing chronic pain? *Select all that apply.*

- Colleagues
- Your own clinical experience
- Patients
- Professional organizations
- Researchers
- Clinical experts
- Pharmaceutical representatives
- Primary peer-reviewed literature (e.g., PubMed)
- Online peer reviewed clinical resources (e.g., UptoDate)
- Email listserv
- Practice Briefs or Practice Guidelines
- Annual conferences
- Seminars at my clinic/institution (e.g., grand rounds; case conference)
- Web-based continuing education modules
- Workshops on specific intervention (e.g. CBT, Yoga)
- Main-stream media (e.g. NPR, CNN, FoxNews)
- Blogs (e.g. Tumblr, Wordpress)
- Social media (e.g. Facebook, Twitter, Reddit)
- Podcasts
- Other ________________________________________________
- None of these

End of Block: Section 1: Learning about Chronic Pain Management (Diss. Congruence)

Start of Block: Section 2: Managing Chronic Pain (Impl. Congruence)

4.1 Section 2: Managing Chronic Pain

You will be presented with several chronic pain management strategies.

4.2   **Currently,** what chronic pain management strategies do you use? *Select all that apply.*

- Conduct a chronic pain needs assessment (i.e., assess barriers to treatment in your clinic)
- Provide targeted support for clinicians treating chronic pain (e.g., clinical supervision, technical assistance)
- Tailor treatments to meet the needs of your patient population (e.g., provide telehealth for rural patients with chronic pain)
- Develop a chronic pain champion in clinic (a local clinic member who is passionate about improving chronic pain management).
- Seek consultation from chronic pain experts
- Develop an interdisciplinary workgroup to address chronic pain
- Provide chronic pain education for patients or caregivers
- Directly engage patients or families in the process of quality improvement around chronic pain management
- Change payment or reimbursement structures for chronic pain management
- Change medical records (e.g., develop new note templates, clinical reminders)
- Use data to inform care (e.g., track quality indicators, provide clinical data to providers)
- Mandate change (change the rules about prescribing)
- None of these

| Page Break |  |
| --- | --- |

4.3 **In an ideal world,** what chronic pain management strategies would you use? *Select all that apply.*

- Conduct a chronic pain needs assessment (i.e., assess barriers to treatment in your clinic)
- Provide targeted support for clinicians treating chronic pain (e.g., clinical supervision, technical assistance)
- Tailor treatments to meet the needs of your patient population (e.g., provide telehealth for rural patients with chronic pain)
- Develop a chronic pain champion in clinic (a local clinic member who is passionate about improving chronic pain management).
- Seek consultation from chronic pain experts
- Develop an interdisciplinary workgroup to address chronic pain
- Provide chronic pain education for patients or caregivers
- Directly engage patients or families in the process of quality improvement around chronic pain management
- Change payment or reimbursement structures for chronic pain management
- Change medical records (e.g., develop new note templates, clinical reminders)
- Use data to inform care (e.g., track quality indicators, provide clinical data to providers)
- Mandate change (change the rules about prescribing)
- None of these
